# Cinnamon-Mediated Silver Nanoparticles and Beta-Carotene Nanocarriers in Alginate Dressings for Wound Healing Applications

**DOI:** 10.3390/gels11090738

**Published:** 2025-09-15

**Authors:** Anca Elena Țăin (Anastasiu), Alexandra Cătălina Bîrcă, Monica Sânziana Nedelcu, Alina Maria Holban, Adelina-Gabriela Niculescu, Alexandru Mihai Grumezescu, Ariana Hudiță

**Affiliations:** 1 Department of Science and Engineering of Oxide Materials and Nanomaterials, National University of Science and Technology Politehnica Bucharest, 011061 Bucharest, Romania; anca_tain@yahoo.com (A.E.Ț.); ada_birca@yahoo.com (A.C.B.); monika.sanziana@yahoo.com (M.S.N.); adelina.niculescu@upb.ro (A.-G.N.); 2 Faculty of Biology, University of Bucharest, 030018 Bucharest, Romania; alina_m_h@yahoo.com (A.M.H.); ariana.hudita@bio.unibuc.ro (A.H.); 3 Research Institute of the University of Bucharest—ICUB, University of Bucharest, 050657 Bucharest, Romania

**Keywords:** alginate-hydrogel, silver-cinnamon nanoparticles, beta-carotene polymeric nanoparticles, centella asiatica, antimicrobial, regenerative

## Abstract

The natural wound healing process is often insufficient to restore tissue integrity in the case of chronic wounds, particularly when skin disruption is accompanied by pathological complications. The severity of these wounds is frequently exacerbated by persistent inflammation and the formation of bacterial biofilms, which significantly hinder skin regeneration. In this study, a pharmaceutical hydrogel-based wound dressing was developed and evaluated, incorporating silver nanoparticles synthesized with cinnamon essential oil that serves as both a stabilizer and antimicrobial agent, polymeric beta-carotene nanoparticles, and *Centella asiatica* extract. The work details the synthesis of both types of nanoparticles, their integration into an alginate-based matrix, and the subsequent formulation of composite dressings. The influence of each therapeutic agent on the morphology and structural characteristics of the dressings was demonstrated, along with the evaluation of their antimicrobial performance against both Gram-positive and Gram-negative bacterial strains. The antimicrobial effects observed within the first 24 h, critical for wound dressing application, highlight the potential of the developed materials for effective chronic wound management. A comprehensive set of analyses was performed to characterize the synthesized nanostructures and the final dressings. These included XRD, FTIR, SEM, EDS, and DLS. Additionally, swelling and degradation tests were conducted to assess hydrogel performance, while antimicrobial and antibiofilm activities were tested against *Staphylococcus aureus* and *Escherichia coli* over a 24-h period. The biocompatibility screening of the alginate-based wound dressings was performed on human keratinocyte cells and revealed that the incorporation of beta-carotene and *Centella asiatica* into alginate-based wound dressings effectively mitigates silver-induced cytotoxicity and oxidative stress and determines the development of highly biocompatible wound dressings. This paper presents an alginate hydrogel co-loaded with Ag nanoparticles, BC@PVP, and *Centella asiatica* extract that balances antimicrobial efficacy with cytocompatibility. Pairing silver with natural antioxidant/anti-inflammatory components mitigates cell stress while retaining broad activity, and the nanoparticle choice tunes pore architecture to optimize moisture and exudate control in chronic wounds.

## 1. Introduction

Chronic wounds can significantly impact patient lifestyle, and they may result not only from traumatic injuries but also as a consequence of underlying chronic conditions such as obesity, diabetes, or venous insufficiency, conditions that are increasingly prevalent in the general population. Unlike acute wounds, which typically heal within 8 to 12 weeks, chronic wounds are clinically defined by their failure to progress through the normal stages of healing, often remaining unhealed beyond three months. At the wound site, a persistent state of inflammation develops due to abnormal cellular and molecular interactions, largely exacerbated by infection and prolonged tissue damage. This pathological environment is marked by a complex interplay of biological factors, including elevated levels of reactive oxygen species (ROS) [1,2,3], impaired angiogenesis [4,5], stalled re-epithelialization [6,7], and critically reduced concentrations of growth factors and cytokines [8,9], all of which hinder the body’s natural repair mechanisms. In this context, infection becomes a major barrier to wound closure and tissue regeneration, with the formation of microbial biofilms being particularly problematic. These biofilms, formed after initial microbial colonization, are notoriously difficult to eradicate and contribute heavily to the chronicity of the wound [5,10,11,12,13,14,15]. Effective management of chronic wounds, therefore, requires comprehensive care strategies involving multiple coordinated steps. Skipping or improperly applying any stage of treatment can significantly delay recovery. Alongside wound care procedures, appropriate therapeutic interventions are essential [16].

Advanced wound dressings have emerged as a critical component of modern wound care, designed to meet the stringent requirements of chronic wound environments. Ideal dressings must not only serve as a physical barrier against external contaminants, given the compromised integrity of the skin, but also maintain a moist wound environment, absorb excess exudate, allow gas exchange through porosity, and provide anti-inflammatory and analgesic effects. Moreover, antimicrobial properties are essential, alongside ensuring biocompatibility, biodegradability, and non-allergenicity [17,18,19,20,21,22].

Recent clinical and preclinical studies have emphasized that these multifunctional features directly contribute to improved healing outcomes in chronic wounds when compared with conventional dressings such as gauze [23,24,25,26,27].

Therefore, the human body possesses an intrinsic ability to initiate complex healing processes in response to injury. However, in certain pathological conditions, such as chronic wounds, this capacity becomes severely impaired. Chronicity can overwhelm the body’s natural regenerative mechanisms, particularly when infections, necrotic tissue, and persistent inflammation are involved. In such cases, external intervention is essential to stimulate and support tissue regeneration and restore the physiological balance. Modern advancements in biomedical engineering have enabled the refinement of traditional healing materials through the integration of nanotechnology and targeted synthesis strategies. These developments involve the use of common yet biologically compatible compounds, re-engineered to exhibit enhanced physicochemical properties that elicit favorable biological responses [28,29,30,31].

Various strategies have been explored for chronic wound treatment. For instance, topical antimicrobials are effective in reducing low-grade contamination but may not suffice in controlling established infections, and their prolonged use beyond two weeks is generally discouraged. Iodine, widely used in clinical settings, shows notable antimicrobial activity but may pose allergenic risks. Hypochlorous acid and chlorhexidine also offer broad-spectrum antibacterial efficacy. A key requirement in the selection of antimicrobial agents is that they must selectively target bacterial cells without compromising the viability of host skin cells. Recently, silver nanoparticle-based dressings have gained increasing clinical attention due to their potent and broad-spectrum activity against biofilm-forming pathogens, representing a promising advancement in chronic wound therapy [32,33,34,35,36,37]. Importantly, several studies highlight their effectiveness against biofilm-forming pathogens [38,39,40], as well as against drug-resistant bacteria such as Methicillin-resistant *Staphylococcus aureus* (MRSA) and *Pseudomonas aeruginosa* [40,41,42,43]. This dual action not only prevents colonization but also addresses one of the major clinical challenges in chronic wound management, thereby strengthening their therapeutic relevance.

**Figure 1:**
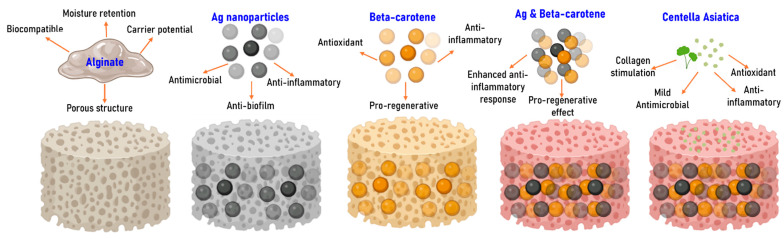
Schematic representation of the wound dressing formulations, highlighting the functional role of each incorporated component in contributing to the overall antimicrobial and regenerative performance.

This study employs silver nanoparticles (Ag), widely recognized for their antimicrobial efficacy against prokaryotic and biocompatible relationship with eukaryotic cells [44,45,46,47,48,49,50]. In this study, Ag nanoparticles are synthesized using a green approach that incorporates D-glucose as a reducing agent and cinnamon essential oil as a stabilizer and synergistic antimicrobial. This synthesis route yields small, uniform particles that maintain strong antimicrobial activity while also promoting biocompatibility due to the organic nature of the capping agents. Complementarily, beta-carotene, a natural antioxidant and pro-regenerative molecule, was selected for its potential to enhance tissue repair [51,52,53,54,55]. By encapsulating beta-carotene in a polyvinylpyrrolidone polymeric nanoparticle matrix (BC@PVP), its stability and release profile are optimized, allowing for sustained therapeutic effects and improved tolerance by the host tissue. These therapeutic agents were integrated into sodium alginate-based hydrogels, a biocompatible polymer known for its non-irritating nature and ability to form porous structures that support tissue oxygenation and healing [56,57,58,59]. The resulting hydrogels not only provide a structural matrix to retain and release the nanoparticles, but also mimic the moist environment required for optimal wound healing. To further boost the bioactivity of the formulation, *Centella asiatica* extract was included for its well-documented wound healing, anti-inflammatory, and antimicrobial properties [60,61,62,63,64]. Each component in the system plays a functional role in the regenerative process, and their mutual compatibility ensures an efficient, cohesive healing response (Figure 1). However, this naturally inspired yet technologically advanced design demonstrates a promising strategy for the development of next-generation wound dressings that employ common, safe materials with upgraded functionality tailored for chronic wound therapy.

## 2. Results and Discussion

The results are presented in the order corresponding to the sequential development of each material. Accordingly, the first part of this section includes the physicochemical characterization data of the Ag nanoparticles and the BC@PVP polymeric nanoparticles.

### 2.1. Silver (Ag) and Polymeric Beta-Carotene (BC@PVP) Nanoparticles Characterization

**Figure 2:**
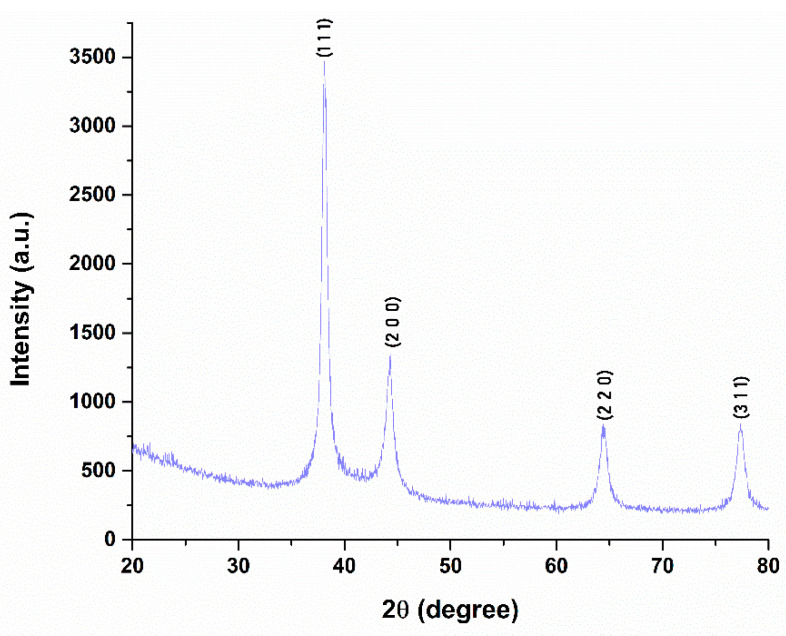
X-ray diffractogram obtained for Ag nanoparticles.

X-ray diffraction analysis was employed to investigate the crystalline structure and lattice parameters of the synthesized silver nanoparticles, as shown in Figure 2. The diffractogram exhibits four well-defined peaks located at 2θ values of 38.0498°, 44.3127°, 64.4480°, and 77.2600°, which correspond to the (111), (200), (220), and (311) crystallographic planes of metallic silver. These reflections are consistent with the standard diffraction data provided by the PDF-ICDD database (reference code 01-077-6577), confirming the formation of pure face-centered cubic (FCC) silver with a space group of Fm-3m. The average crystallite size was calculated using the Debye–Scherrer equation, yielding a value of 4.88 nm, suggesting the formation of nanoscale, crystalline silver particles.

**Figure 3:**
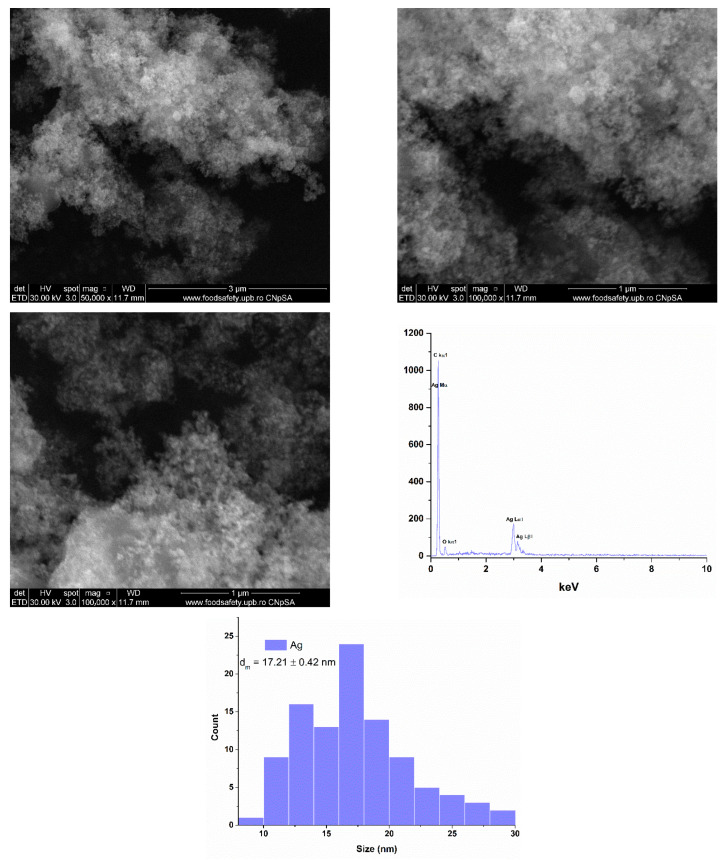
SEM micrographs, EDS data, and particle size distribution obtained for Ag nanoparticles.

The resulting powder was further analyzed by scanning electron microscopy (SEM) to evaluate the morphology and size of the silver nanoparticles. Figure 3 displays the micrographs obtained using a secondary electron detector, revealing predominantly spherical morphologies and a pronounced tendency toward particle agglomeration. Energy-dispersive X-ray spectroscopy (EDS) confirmed the presence of silver as the main element component of the sample, along with carbon (C) and oxygen (O), which are attributed to the cinnamon oil used during the synthesis. The average particle size was determined to be 17.21 ± 0.42 nm, with the presence of cinnamon oil contributing to the stabilization of the nanoparticles and enhancing the reduction process of silver ions.

**Figure 4:**
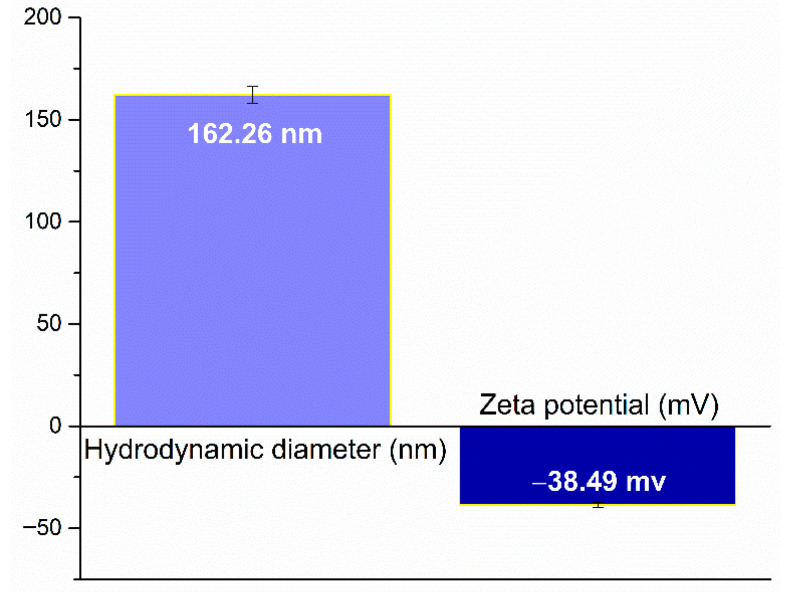
DLS analysis of Ag nanoparticles, presenting the hydrodynamic diameter and zeta potential.

Dynamic light scattering (DLS) analysis was employed to assess the hydrodynamic diameter and zeta potential of the Ag nanoparticles, providing insight into their physicochemical behavior in suspension. The results are illustrated in Figure 4. The measured hydrodynamic diameter was approximately 162.26 nm, indicating the average size of the nanoparticles agglomerations in their solvated state, correlating with the SEM micrographs. The zeta potential was determined to be −38.49 mV, a value which suggests a high degree of colloidal stability due to strong electrostatic repulsion between particles.

**Figure 5:**
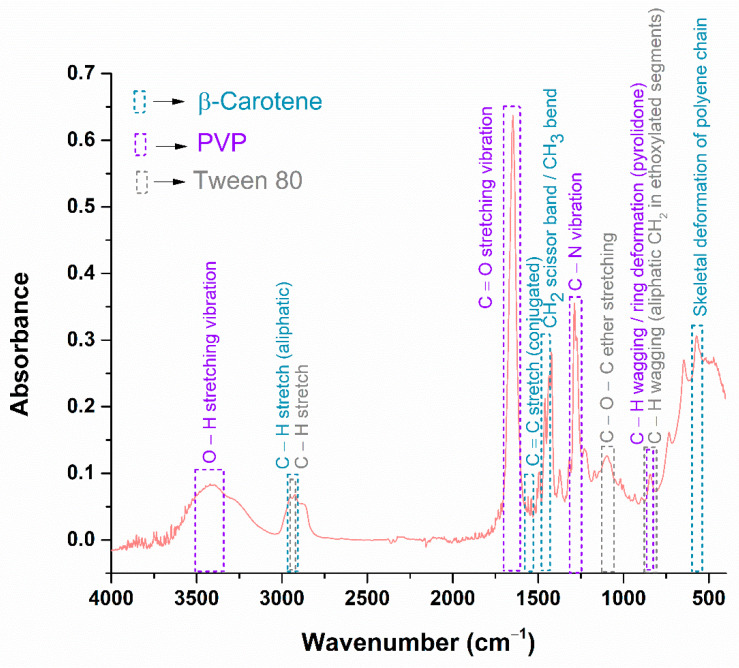
FTIR spectra recorded for BC@PVP polymeric nanoparticles.

The physicochemical characteristics of the silver nanoparticles have been investigated, and the next step involves the characterization of the BC@PVP polymeric nanoparticles, in order to obtain a comprehensive understanding of the therapeutic agents prior to their incorporation into the hydrogel formulations. Accordingly, Figure 5 presents the FTIR spectrum obtained for the BC@PVP sample.

The FTIR analysis highlights the presence of functional groups specific to each component used in the synthesis of BC@PVP polymeric nanoparticles. Figure 5 displays the absorption bands color-coded for beta-carotene, PVP, and Tween 80 to facilitate clearer visualization of their individual contributions. The presence of beta-carotene is primarily indicated by the absorption band at 1436 cm^−1^, a well-known fingerprint region attributed to CH_2_ scissoring and CH_3_ asymmetric deformation vibrations. Another characteristic absorption band is observed at 571 cm^−1^, associated with skeletal deformation of the polyene chain, which reflects the conjugated backbone of beta-carotene. Additionally, the band at 1558 cm^−1^ corresponds to the C=C stretching vibration of the conjugated double bonds in beta-carotene. However, due to the presence of PVP and Tween 80 in the nanoparticle system, most of the beta-carotene characteristic bands are overlapped by the more intense bands of the polymeric and surfactant components. PVP is identified by the C–N stretching vibration at 1287 cm^−1^, the C=O stretching band at 1647 cm^−1^ (from the pyrrolidone ring), and the C–H wagging mode at 844 cm^−1^, associated with ring deformation. Tween 80 also contributes to the band at 844 cm^−1^ through C–H wagging vibrations of aliphatic CH_2_ groups in its ethoxylated chains. Moreover, the absorption at 1096 cm^−1^ corresponds to the C–O–C ether stretching, which is characteristic of Tween 80. Table 1 summarizes the FTIR analysis results in tabular form.

**Figure 6:**
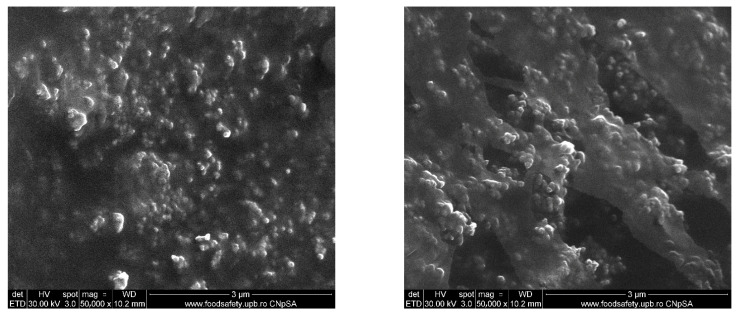

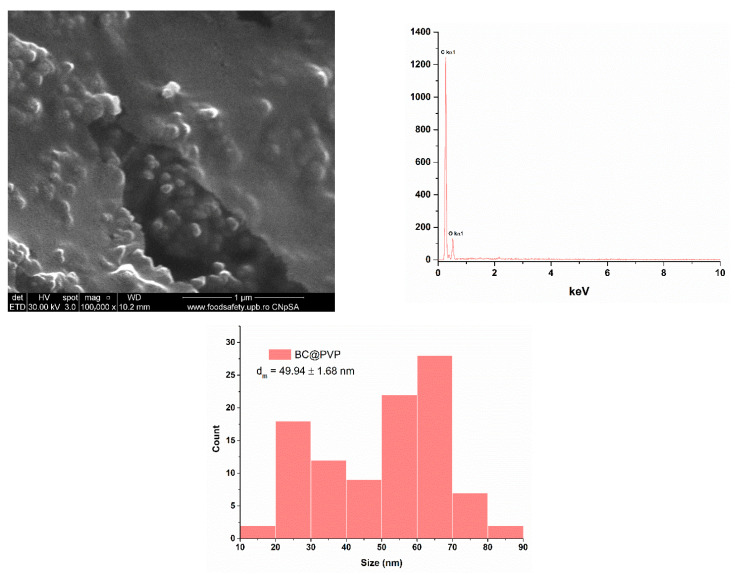
SEM micrographs and EDS results obtained for BC@PVP polymeric nanoparticles.

BC@PVP nanoparticles were further analyzed to investigate their morphology, elemental composition, and particle size using scanning electron microscopy. As shown in Figure 6, numerous polymeric particles with predominantly spherical morphology were observed, uniformly distributed and clearly distinguishable as individual nanoparticles. The EDS analysis confirmed the elemental composition consistent with the synthesis process, with carbon (C) and oxygen (O) being identified. Particle size distribution analysis revealed an average diameter of 49.94 ± 1.68 nm. The particles displayed a monomodal and relatively uniform size distribution, consistent with the nanoscale nature of the system.

**Figure 7:**
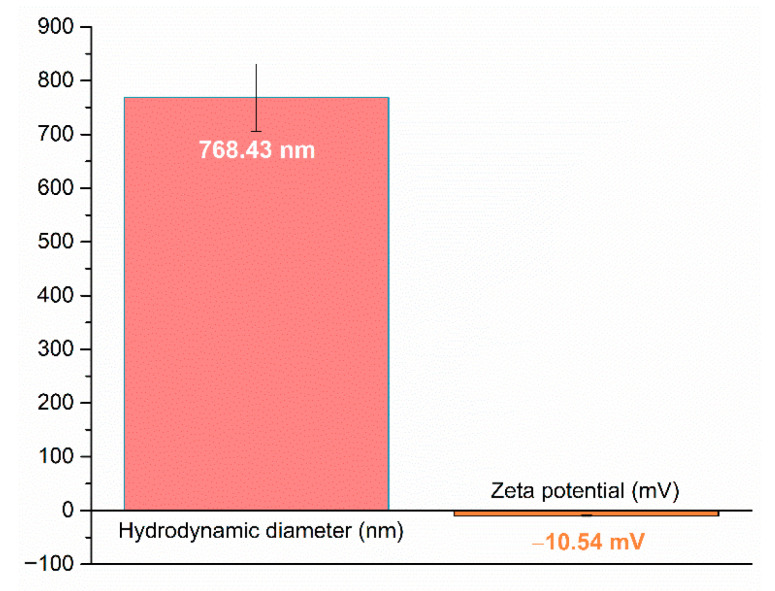
DLS analysis of BC@PVP polymeric nanoparticles, presenting the hydrodynamic diameter and zeta potential.

The BC@PVP polymeric nanoparticles were characterized using dynamic light scattering to evaluate their hydrodynamic diameter and zeta potential. Figure 7 presents the graphical representation of the obtained data. The measured hydrodynamic diameter was 768.43 nm, indicating a clear tendency of agglomeration regarding the physical size calculated from SEM micrographs, while the zeta potential was −10.54 mV, indicating a moderate colloidal stability of the BC@PVP nanoparticles in suspension.

In summary, Ag nanoparticles were successfully synthesized, with silver identified as the only crystalline phase in the XRD diffractogram. Their spherical morphology and nanoscale physical dimensions were confirmed by SEM micrographs. Although DLS analysis indicated a strong tendency toward particle aggregation, the AgNPs maintained high stability even in their agglomerated state. Similarly, the BC@PVP polymeric nanoparticles exhibited characteristic functional groups as confirmed by FTIR spectroscopy, along with spherical morphology and clear identification of individual particles in SEM images. Their physical dimensions remained within the nanometric scale, while the hydrodynamic diameter was significantly higher due to particle aggregation, which is consistent with the moderate colloidal stability observed in suspension.

In the next stage, the synthesized Ag nanoparticles and BC@PVP polymeric nanoparticles were incorporated into alginate-based wound dressing formulations, where they serve as therapeutic agents to support the healing process of chronic wounds. Section 2.2 presents the results obtained for the five developed alginate-based wound dressings.

### 2.2. Alginate-Based Wound Dressings Characterization

**Figure 8:**
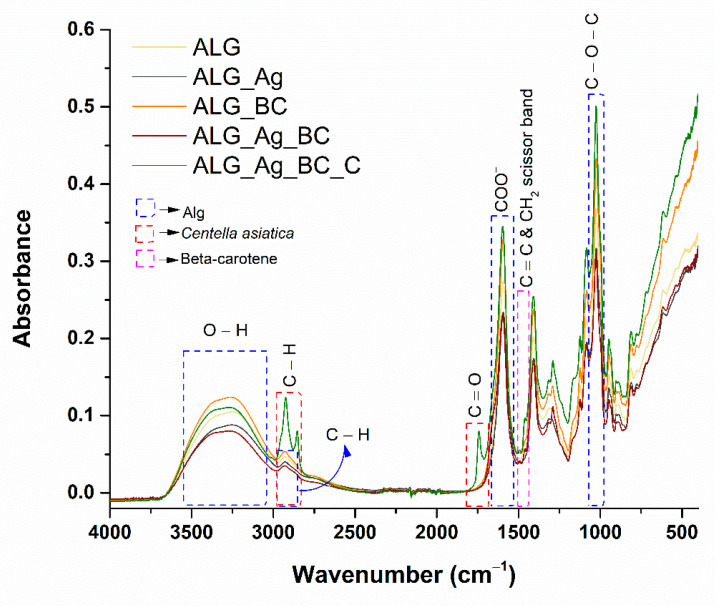
FTIR spectra obtained for ALG, ALG_Ag, ALG_BC, ALG_Ag_BC, and ALG_Ag_BC_C wound dressings.

The successful integration of therapeutic agents into the polymeric matrix was confirmed through FTIR spectroscopy analysis of the developed wound dressings. The resulting spectra, presented in Figure 8, highlight the characteristic absorption bands corresponding to the incorporated compounds.

Due to the predominance of alginate in the developed formulations, the main functional groups identified across all wound dressings are associated with the alginate matrix. The hydrophilic character of the samples is evidenced by the broad absorption band around 3200 cm^−1^, corresponding to hydroxyl groups. The peak at 2927 cm^−1^ is attributed to C–H symmetric stretching, while the absorption at 1593 cm^−1^ corresponds to the asymmetric and symmetric stretching vibrations of the carboxylate (COO^−^) group. Additionally, the band at 1024 cm^−1^ is associated with C–O–C asymmetric stretching. The most distinct evidence of BC@PVP polymeric nanoparticles is observed at 1491 and 1458 cm^−1^, attributed to the C=C stretching vibration and CH_2_ scissoring vibration bands of beta-carotene. These bands are present exclusively in the ALG_BC, ALG_Ag_BC, and ALG_Ag_BC_C dressings. Regarding the incorporation of *Centella asiatica*, successful integration into the ALG_Ag_BC_C formulation is confirmed by the distinct spectral differences compared to the other samples. The absorption bands at 2924 and 2854 cm^−1^ are associated with C–H stretching vibrations originating from amino acids found in *Centella asiatica* (glutamic acid, arginine, histidine, tyrosine) and natural lipids known as phytosterols (sitosterol and stigmasterol). Furthermore, the absorption at 1741 cm^−1^ corresponds to the C=O stretching vibration typical of pectin, lignin, and hemicellulose, also present in the *Centella asiatica* extract. No significant shifts in characteristic band positions were observed after the incorporation of Ag or BC@PVP nanoparticles. Instead, only changes in band intensity and broadening were detected, consistent with physical entrapment of the nanoparticles in the alginate network rather than the formation of new covalent bonds.

**Figure 9:**
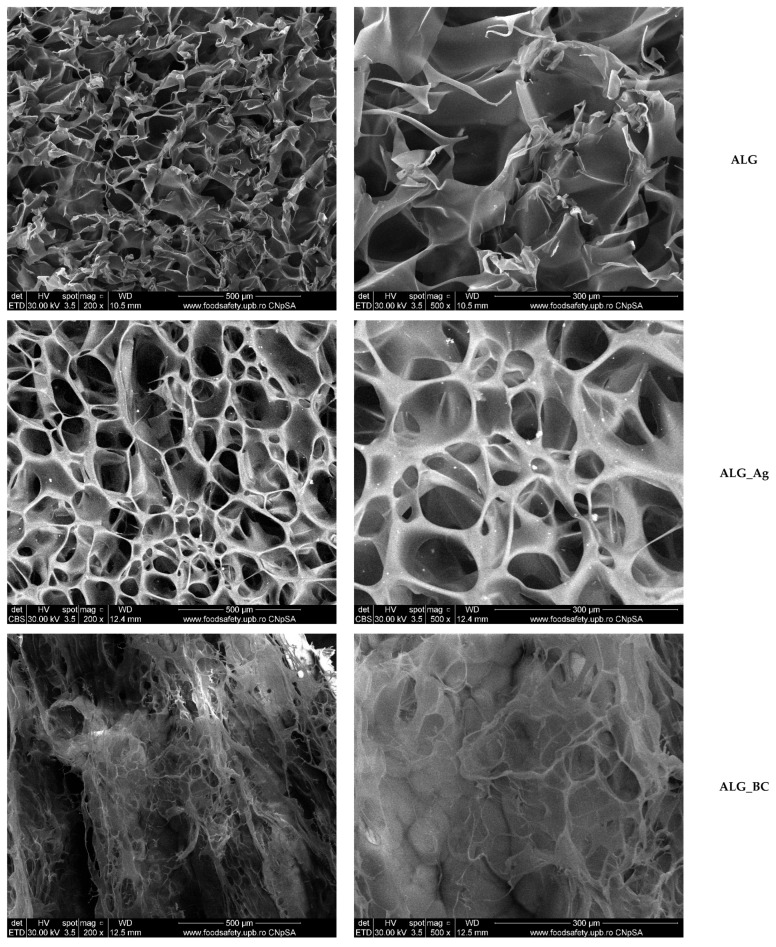

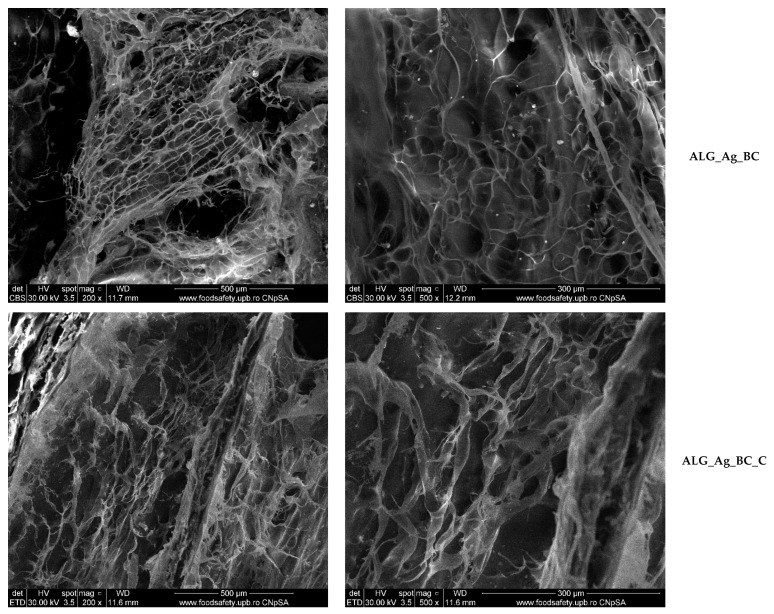
SEM micrographs obtained for ALG, ALG_Ag, ALG_BC, ALG_Ag_BC, ALG_Ag_BC_C wound dressings.

Figure 9 presents SEM micrographs of the five alginate-based wound dressings (ALG, ALG_Ag, ALG_BC, ALG_Ag_BC, and ALG_Ag_BC_C) recorded at magnifications of X200 and X500. Micrographs for ALG, ALG_BC, and ALG_Ag_BC_C were acquired using the secondary electron detector, while ALG_Ag and ALG_Ag_BC samples were analyzed using backscattered electron mode in order to better visualize the distribution of Ag nanoparticles.

The control sample (ALG), consisting only of the alginate matrix, exhibits a highly porous structure with uneven pore distribution and partial collapse of the internal architecture, likely due to disordered polymeric layering. In contrast, the ALG_Ag sample shows a more cohesive porous network with better-defined and interconnected pores. This structural improvement is attributed to the incorporation of Ag nanoparticles, which appear uniformly distributed throughout the matrix and contribute to pore stability, as revealed in backscattered SEM micrographs.

The addition of BC@PVP nanoparticles in the ALG_BC formulation also led to significant morphological changes. While numerous pores are visible, the supporting polymeric structure appears more fragile and less interconnected compared to ALG_Ag, indicating that the polymeric nanoparticles influence the network without reinforcing it to the same degree. The ALG_Ag_BC sample exhibits a hybrid morphology, combining features of both ALG_Ag and ALG_BC. The pore network is visibly more stable than in ALG_BC, reflecting the structural reinforcement provided by Ag nanoparticles, while maintaining the general architecture observed in BC-loaded samples. The silver nanoparticles remain well-distributed, contributing to overall matrix integrity. The final formulation, ALG_Ag_BC_C, includes *Centella asiatica* extract as a botanical healing enhancer. SEM analysis shows that the porous morphology is preserved, with no significant structural differences compared to ALG_Ag_BC. The continuity of the porous framework and the uniformity of the internal structure suggest that the inclusion of the botanical extract does not interfere with the hydrogel’s physical integrity.

**Figure 10:**
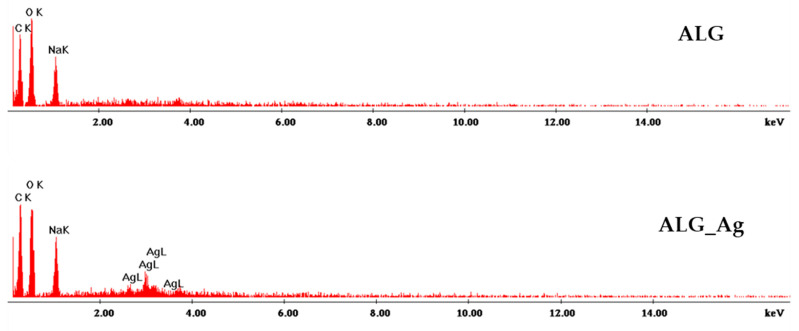

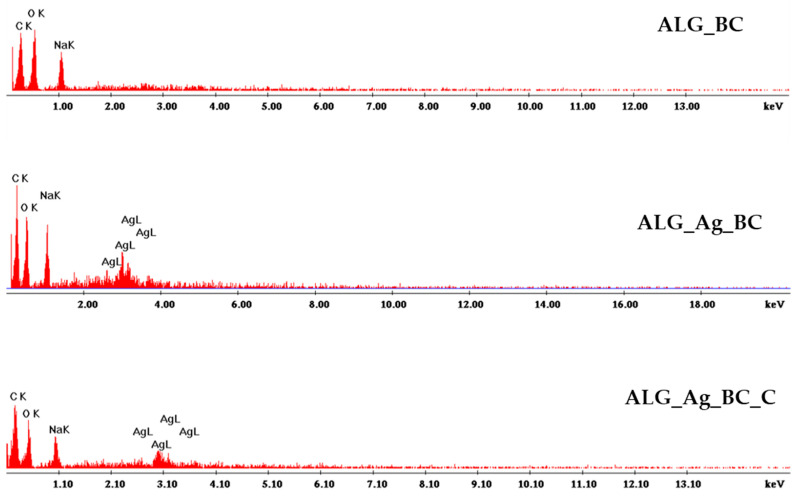
EDS spectra obtained for ALG, ALG_Ag, ALG_BC, ALG_Ag_BC, and ALG_Ag_BC_C wound dressings.

The elemental composition of each dressing was examined using the SEM-EDS module, and the results are presented in Figure 10. Because alginate constitutes the matrix of all formulations, carbon, oxygen, and sodium signals are prominent. For dressings incorporating silver nanoparticles, the presence of silver is confirmed by the characteristic Ag peaks in the EDS spectra.

**Figure 11:**
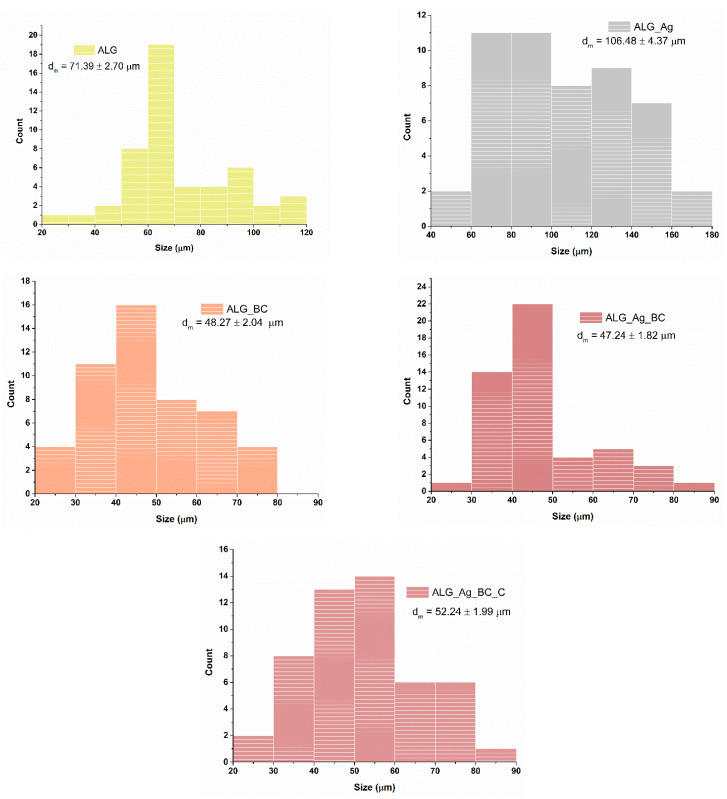
ALG, ALG_Ag, ALG_BC, ALG_Ag_BC, and ALG_Ag_BC_C dressings’ pore size distribution.

Figure 11 illustrates the pore size distribution of the five alginate-based wound dressings. The control sample (ALG) displayed a broad and irregular distribution, with an average pore size of 71.39 ± 2.70 μm, reflecting a less organized internal structure. The addition of Ag nanoparticles (ALG_Ag) resulted in the largest pores (106.48 ± 4.37 μm), likely due to their influence on the gelation and freezing processes. In contrast, BC@PVP incorporation (ALG_BC) reduced the average pore size to 48.27 ± 2.04 μm, possibly by increasing matrix density. The dual-loaded formulation (ALG_Ag_BC) showed the smallest and most uniform pores (47.24 ± 1.82 μm), suggesting a synergistic structural effect. The inclusion of *Centella asiatica* extract (ALG_Ag_BC_C) slightly increased the pore size to 52.24 ± 1.99 μm, without compromising the overall porosity.

**Figure 12:**
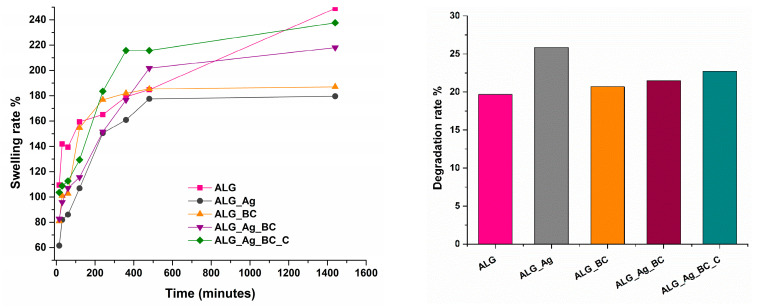
Time-dependent swelling and degradation rate of the ALG, ALG_Ag, ALG_BC, ALG_Ag_BC, and ALG_Ag_BC_C wound dressings after 24 h.

The time-dependent swelling capacity of all five wound dressing formulations was evaluated and is illustrated in Figure 12.

The lowest swelling rate over the 24-h period was observed for the ALG_Ag sample, which can be attributed to its larger pore network and the partial pore blockage caused by the presence of silver nanoparticles. The ALG_Ag_BC dressing shows a swelling profile comparable to that of ALG_Ag_BC_C, indicating that the addition of the botanical extract does not significantly affect this property of the dressings. Overall, all formulations exhibit swelling ratios exceeding 100%, confirming their ability to effectively absorb wound exudate.

The degradation behavior of the hydrogels was evaluated over a 24-h period, with the results shown in Figure 11. Among the five formulations, ALG_Ag exhibited the highest degradation rate, which may be attributed to the release of silver nanoparticles and the subsequent reduction in the overall mass of the dressing. In contrast, the dressings containing BC@PVP polymeric nanoparticles demonstrated similar and more moderate degradation rates, likely due to the stabilizing interactions between the polymers and the comparable morphological characteristics of these formulations.

**Figure 13:**
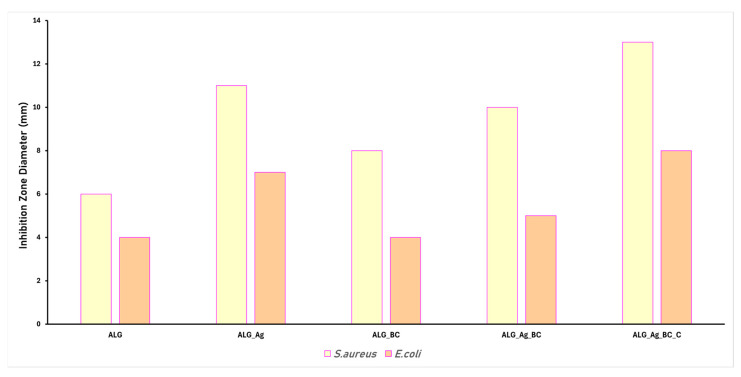
Inhibition zone diameters (cm) for *S. aureus* and *E. coli* after 24 h of incubation with the obtained dressings (ALG, ALG_Ag, ALG_BC, ALG_Ag_BC, and ALG_Ag_BC_C).

**Figure 14:**
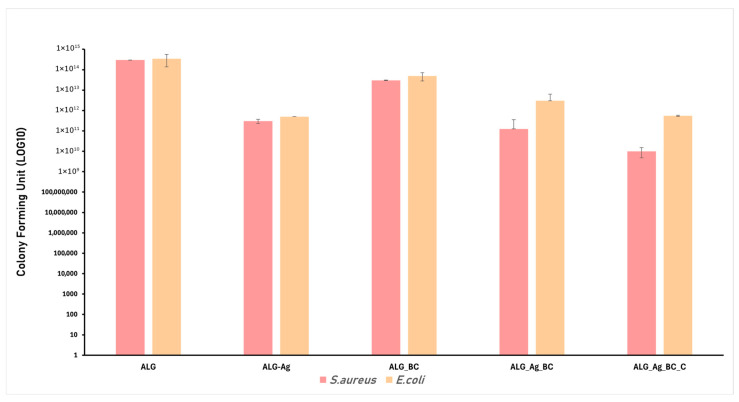
Graphical representation of biofilm modulation after 24 h of dressing exposure on *S. aureus* and *E. coli*.

Chronic wounds are often associated with severe infections that significantly delay the natural healing process. Modern wound dressing development increasingly focuses on incorporating antimicrobial properties through the addition of active agents. In this study, Ag nanoparticles were included as antimicrobial components, prepared by a green method. Based on this strategy, the alginate-based formulations, ALG, ALG_Ag, ALG_BC, ALG_Ag_BC, and ALG_Ag_BC, were evaluated for their antimicrobial performance against *Staphylococcus aureus* and *Escherichia coli*, two of the most common and clinically relevant bacterial strains found in infected wounds. The results of the antimicrobial assays are graphically presented below, including inhibition zone diameter measurements (Figure 13) and biofilm modulation (Figure 14) after 24 h of incubation.

The inhibition zone diameter (IZD) test results support the theoretical antimicrobial potential of each material used in the wound dressing formulations. Compared to the control sample (ALG), which lacks inherent antimicrobial properties, a clear enhancement was observed upon the addition of silver nanoparticles. Specifically, the ALG_Ag dressing showed IZDs of 12 mm against *S. aureus* and 7 mm against *E. coli*. The ALG_BC formulation displayed slightly lower antibacterial efficiency than ALG_Ag, yet outperformed the control, particularly against *S. aureus*. The dual-loaded dressing (ALG_Ag_BC) exhibited a larger IZD than ALG_BC, but was slightly smaller than ALG_Ag, indicating that the co-loaded formulation retains strong antibacterial efficacy against both tested strains. Notably, the ALG_Ag_BC_C formulation, which includes *Centella asiatica* extract, exhibited the highest antimicrobial activity against both bacterial strains, suggesting that the botanical additive enhances and supports the antimicrobial effectiveness of the dressing.

The effect of the developed wound dressings on biofilm modulation was assessed by quantifying the number of viable bacteria (expressed as CFU/mL in log in base 10 scale) after 24 h of incubation with *S. aureus* and *E. coli*. The control sample (ALG) showed no inhibitory effect, as evidenced by extremely high CFU values (above 10^14^) for both bacterial strains. In contrast, the incorporation of Ag nanoparticles in the ALG_Ag dressing resulted in a drastic reduction in bacterial viability, particularly against *S. aureus*, confirming the strong antibiofilm activity of Ag nanoparticles. The ALG_BC sample exhibited moderate biofilm inhibition, more evident for *S. aureus*, while having a limited impact on *E. coli*, suggesting a strain-dependent effect of the BC@PVP nanoparticles. The dual-loaded formulation (ALG_Ag_BC) showed improved biofilm reduction compared to the individual components, supporting a cumulative effect between Ag nanoparticles and BC@PVP. In the ALG_Ag_BC_C formulation, each component serves a specific role: Ag nanoparticles provide antimicrobial activity, while BC@PVP nanoparticles and *Centella asiatica* extract are included to support tissue regeneration through antioxidant and anti-inflammatory effects. Consequently, the Ag nanoparticles-only dressing is expected to show the strongest antibacterial efficacy. By contrast, in the co-loaded system, interactions between Ag nanoparticles and BC@PVP can enhance stabilization and more homogeneous dispersion of silver [72,73,74], but the presence of β-carotene and *Centella asiatica* may moderate the oxidative component of silver bactericidal action [75,76]. The most substantial biofilm inhibition was observed for the ALG_Ag_BC_C sample, which combined both nanotherapeutics and *Centella asiatica* extract. This sample demonstrated the lowest CFU values for both bacterial strains, highlighting the potential added value of the botanical extract in enhancing antibiofilm performance.

**Figure 15:**
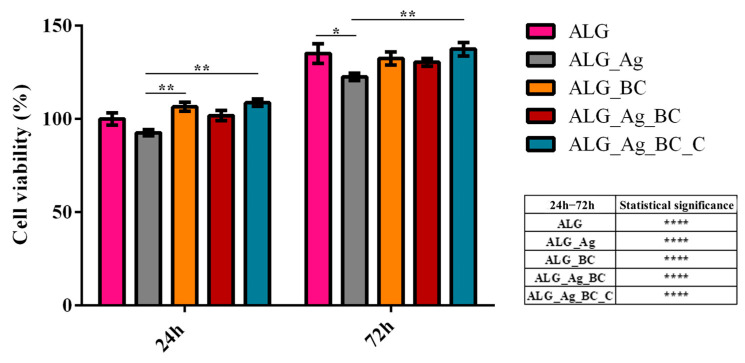
Cell viability of human HaCaT keratinocytes after 24 and 72 h of culture on alginate-based wound dressings. All experiments were performed in triplicate, and the results presented represent the mean of three independent experiments. Statistical significance: * *p* ≤ 0.05, ** *p* ≤ 0.01, **** *p* ≤ 0.0001.

Assessing the biocompatibility of the developed wound dressing materials in essential to ensure their safety and suitability for potential biomedical applications. To this end, HaCaT keratinocytes were cultured in contact with the alginate-based wound dressings for 24 and 72 h, after which cell viability was assessed using the 3-(4,5-dimethylthiazol-2-yl)-2,5-diphenyl tetrazolium bromide (MTT) assay (Figure 15).

After 24 h of culture, HaCaT cells cultured on all dressing formulations exhibited cell viability levels comparable to those cultured on the pristine alginate control (ALG). However, a slight decrease in viability was observed for the ALG_Ag group compared to ALG, although this reduction was not statistically significant, suggesting only a mild cytotoxic effect from silver ions at early exposure. Interestingly, the ALG_Ag_BC_C group showed a statistically significant increase in cell viability compared to ALG_Ag, indicating that the incorporation of beta-carotene and *Centella asiatica* effectively mitigates the silver-induced cytotoxicity.

After 72 h of culture, all experimental groups revealed a statistically significant increase in cell viability compared to their cell viability profile determined after 24 h, indicating that all formulations support human keratinocytes proliferation over time. Moreover, the cell viability of HaCaT cells after 72 h of culture for all samples was similar to the cell viability of human keratinocytes cultured on pristine ALG dressing, except for Alg_Ag. The ALG_Ag group presented a statistically significant decrease in viability, highlighting a persistent cytotoxic effect likely attributable to prolonged silver exposure. Importantly, the ALG_Ag_BC_C formulation exhibited the highest cell viability after 72 h of culture, suggesting a synergistic effect between the beta-carotene and *Centella asiatica* in counteracting the cytotoxic stress induced by silver.

**Figure 16:**
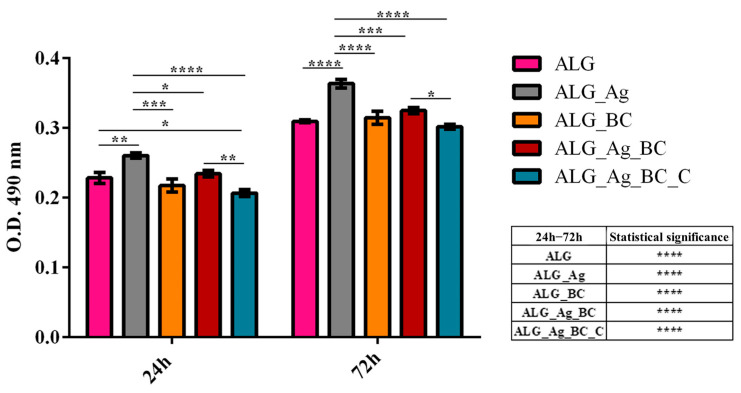
Quantification of LDG release from HaCaT keratinocytes cultured on alginate-based wound dressings after 24 and 72 h. All experiments were performed in triplicate, and the results presented represent the mean of three independent experiments. Statistical significance: * *p* ≤ 0.05, ** *p* ≤ 0.01, *** *p* ≤ 0.001, **** *p* ≤ 0.0001.

To complement the MTT assay and further characterize the cytotoxic potential of the wound dressing formulations, Lactate Dehydrogenase (LDH) leakage in the culture medium was measured after 24 and 72 h of HaCaT cell culture in contact with the developed alginate-based wound dressings (Figure 16). After 24 h of culture, human keratinocytes cultured on ALG_Ag exhibited a significantly higher LDH release profile compared to the control ALG group, indicating early membrane damage likely induced by the presence of silver ions. In contrast, ALG_BC and ALG_Ag_BC induced a similar LDH release profile with the pristine ALG control, suggesting low cytotoxicity for these samples at this time point. Interestingly, human keratinocytes cultured on ALG_Ag_BC_C presented a statistically significant reduction in LDH release compared to ALG control dressing, revealing the lack of cytotoxic effect of this formulation. Moreover, a statistically significant reduction in LDH release for HaCaT cells cultured on ALG_Ag_BC_C was observed also in comparison with ALG_Ag, reinforcing the protective role of beta-carotene and *Centella asiatica* in minimizing silver-induced damage.

After 72 h, LDH levels increased in all groups as compared with the results obtained after 24 h, as expected due to extended culture time. At this time point, the ALG_Ag group maintained the highest LDH release among all samples, further confirming persistent cytotoxic effects. Moreover, ALG_BC and ALG_Ag_BC showed similar LDH levels to the experimental control dressing ALG, while ALG_Ag_BC_C showed significantly lower LDH release than ALG_Ag, similar to the control ALG group, suggesting effective cytoprotection over prolonged exposure. The obtained results align with the MTT assay results, demonstrating that while silver triggers a mild cytotoxic stress for human keratinocytes, its effects can be mitigated by the inclusion of antioxidant and bioactive agents such as beta-carotene and *Centella asiatica,* compounds that preserve human keratinocytes’ membrane integrity and enhance the overall biocompatibility of the developed alginate-based wound dressings.

**Figure 17:**
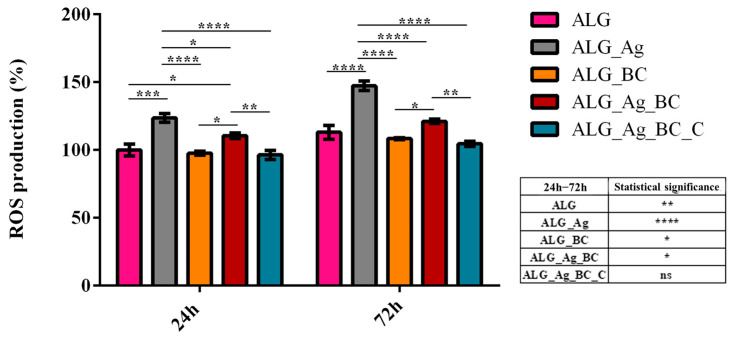
Quantification of intracellular reactive oxygen species (ROS) in HaCaT keratinocytes cultured on alginate-based wound dressings after 24 and 72 h. All experiments were performed in triplicate, and the results presented represent the mean of three independent experiments. Statistical significance: * *p* ≤ 0.05, ** *p* ≤ 0.01, *** *p* ≤ 0.001, **** *p* ≤ 0.0001.

To further investigate the potential oxidative stress induced by the wound dressing formulations, intracellular ROS levels were quantified after 24 and 72 h of HaCaT contact with the alginate-based wound dressings (Figure 17). For the ALG_Ag samples, a statistically significant increase in ROS production was observed at both experimental time points as compared with the experimental control (ALG), indicating that silver ions induce oxidative stress in human keratinocytes. In contrast, ALG_BC and ALG_Ag_BC_C samples presented a similar ROS production pattern with the control ALG at both time points, showing that samples do not trigger oxidative stress in human keratinocyte culture. Moreover, for HaCaT cells cultured on ALG_Ag_BC, the ROS levels were statistically significantly decreased as compared with ALG_Ag at both time points, showing that the addition of beta-carotene in the dressing structure partially attenuates the silver-induced oxidative stress, while levels still remain higher as compared with the ALG sample. Most notably, ALG_Ag_BC_C presented ROS levels statistically comparable to the control at both 24 and 72 h, and showed no significant increase between the two time points, emphasizing the synergic antioxidant protection conferred by beta-carotene and *Centella asiatica*.

### 2.3. Discussion

The experimental results presented in this study highlight the successful development and comprehensive characterization of multifunctional alginate-based wound dressings incorporating green-synthesized silver nanoparticles (Ag) and beta-carotene-loaded polymeric nanocarriers (BC@PVP). Each component was carefully selected for its biocompatibility and therapeutic potential, aiming to address the multifaceted pathology of chronic wounds. The findings confirm not only the efficient integration of these therapeutic agents into the alginate matrix but also their individual and combined contributions to the structural integrity, porosity, antimicrobial performance, biocompatibility, and cell-supportive properties of the final materials.

Chronic wounds pose a major clinical challenge due to impaired healing and a high risk of infection. In this context, the development of advanced biomaterials that integrate antimicrobial, antioxidant, and regenerative properties is of critical importance. The strategy applied in this study by combining silver nanoparticles, beta-carotene, and *Centella asiatica* extract into a biocompatible alginate scaffold represents a nature-inspired yet technologically refined approach to modern wound care. The following discussion explores the relevance of the physicochemical and biological findings in relation to current literature and assesses the potential of the developed formulations as next-generation wound dressings.

The synthesis of Ag nanoparticles in this study was a key process, considering the multiple factors that can influence their physicochemical properties depending on the substances involved. The reducing agent used was D-glucose, a common sugar compound naturally produced by plants through photosynthesis. In order to promote nucleation and the formation of a large number of nanoparticles, NaOH provided the necessary alkalinity for the reduction solution. It is also known that the use of D-glucose in Ag nanoparticle synthesis typically leads to the formation of smaller nanoparticles and, implicitly, a higher tendency for aggregation [77]. Additionally, maintaining a temperature above 80 °C ensures a balanced interplay between the nucleation and growth processes during synthesis. Given these conditions, cinnamon essential oil was included in the synthesis, as its phytoconstituents (particularly cinnamaldehyde and eugenol) play an important role in facilitating the reduction from Ag^+^ to Ag^0^, while also acting as capping agents and contributing to nanoparticle stabilization in suspension [78]. Cinnamaldehyde, the main active compound in cinnamon oil, possesses various biological properties such as analgesic and antiseptic effects, and when used in the synthesis of Ag nanoparticles, it undergoes a redox reaction that results in its conversion into cinnamic acid. This transformation leads to a synergistic effect with the Ag nanoparticles, significantly enhancing their antimicrobial activity [79].

The results obtained from the physicochemical characterization of the Ag nanoparticles confirm the distinct contributions of each reagent used in the synthesis. Crystallinity of the silver nanoparticles was confirmed by X-ray diffraction analysis, where sharp and well-defined peaks corresponding to metallic silver (Ag^0^) were observed, with no additional phases detected. The particle size distribution, determined by SEM analysis, revealed a narrow, monomodal profile with an average diameter of 17.21 ± 0.42 nm, indicating a uniform population of spherical nanoparticles and effective size control during synthesis. Moreover, DLS analysis results indicated a hydrodynamic diameter of 162.26 nm, and the zeta potential measurement revealed a value of −38.49 mV, reflecting high colloidal stability of the nanoparticle suspension. Most green synthesis methods for silver nanoparticles involving cinnamon extract typically rely solely on the silver precursor and the extract itself. However, the physicochemical properties of the resulting nanoparticles can be significantly improved by supplementing the reaction system with additional natural compounds that contribute to nucleation, stabilization, and particle growth. For example, in a study conducted by Y.G. El-Baz et al. [80], silver nanoparticles were synthesized using cinnamon bark extract and silver nitrate solution through continuous stirring at room temperature for 8 h. The DLS results indicated a zeta potential of −12.3 mV with a hydrodynamic size of 201 nm, while SEM analysis revealed roughly spherical, polydisperse nanoparticles with diameters ranging from 6 to 37 nm. Similarly, in a study by J. Premkumar et al. [81], silver nanoparticles were synthesized using cinnamon extract, silver nitrate, and distilled water at various extract-to-metal precursor ratios, followed by incubation at 37 °C for 48 h. The nanoparticles obtained exhibited sizes ranging from 50 to 70 nm.

Beta-carotene is a cyclic carotenoid compound characterized by 11 conjugated double bonds, exhibiting high hydrophobicity and pronounced chemical instability [82]. To enable its effective and stable delivery at the wound site, it was necessary to encapsulate beta-carotene within polymeric carriers. Several formulation techniques have been developed to improve the bioavailability of beta-carotene in medical applications and to achieve submicron particle sizes. These include emulsification-evaporation, high-pressure homogenization, antisolvent precipitation, and hot homogenization. In this study, a simplified version of the hot homogenization method, assisted by ultrasound, was employed. In the absence of an aqueous antisolvent phase, nanoparticle formation was achieved via ultrasound-assisted self-assembly and encapsulation in an ethanolic medium, where beta-carotene was solubilized in the presence of PVP and Tween 80. The rapid energy input provided by probe sonication facilitated efficient nucleation and led to the formation of stable, nanosized particles uniformly dispersed throughout the ethanolic system.

A similar method for the preparation of beta-carotene solid lipid nanoparticles (SLNs) was explored by Ashay Jain et al. [83]. In their study, beta-carotene was incorporated alongside glyceryl monostearate and Gelucire 50/13, which were melted together at temperatures below 70 °C. In parallel, Phospholipid S-100 was solubilized in ethanol, and this ethanolic phase was then mixed with the molten lipid phase. The combined mixture was subsequently added to a pre-heated surfactant solution of Tween 80 and Pluronic F68. The entire formulation was homogenized using a high-shear mixer for 30 min at 10,000 rpm. DLS analysis confirmed particle sizes ranging between 200–400 nm, with zeta potential values between +6 and +10 mV (depending on the concentrations used), while FE-SEM imaging confirmed the nanoparticles’ dimensions within the nanometric range. In another study, Rhyan Prayuddy Reksamunandar et al. [84] proposed a PVP-based encapsulation strategy for beta-carotene using electrospinning. PVP was dissolved in ethanol, followed by the addition of beta-carotene, and the mixture was magnetically stirred for 3 h. The resulting solution was loaded into a syringe and processed using electrospinning, resulting in PVP/beta-carotene nanofiber mats. It was demonstrated that the fiber diameter increased proportionally with the polymer concentration. Wan-Yi Liu et al. [85] also demonstrated that incorporating PVP in the formulation of beta-carotene nanoparticles led to a reduction in particle size, from 209 nm in conventional PLGA nanoparticles to 175 nm in hybrid PLGA–PVP nanoparticles, highlighting the size-reducing effect of PVP in such systems. Fei Sheng et al. [86] reported a formulation of beta-carotene nanoparticles via antisolvent precipitation under high-power ultrasound. In their protocol, beta-carotene was first dissolved in tetrahydrofuran, then added to an aqueous antisolvent phase composed of Pluronic F-108 and water. A microprobe delivering high-intensity ultrasound (20 kHz) was applied for up to 5 min. Ultrasound power was shown to significantly influence the particle size: particles synthesized without sonication had an average size of 241 ± 3.6 nm, whereas ultrasound-treated particles were significantly smaller, at 173 ± 3.9 nm. This size reduction was also visible in SEM images, where non-sonicated particles ranged between 200–300 nm, while sonicated particles showed considerably smaller dimensions.

The successful formation of BC@PVP polymeric nanoparticles was further confirmed by FTIR spectroscopy, which revealed the presence of characteristic functional groups associated with both beta-carotene and PVP components [65,66,67,68,69,70].

Following the successful synthesis and characterization of both Ag nanoparticles and BC@PVP polymeric carriers, the next focus of this study was the integration of these bioactive agents into alginate-based wound dressings and the evaluation of their structural, antimicrobial, and regenerative performance. The spectral modifications observed across all dressing formulations offer strong evidence of the successful incorporation of therapeutic agents and the natural extract into the ALG_Ag_BC_C matrix, thereby supporting the enhanced antimicrobial and regenerative properties of the final dressing [87,88,89].

The advanced field of wound dressing design and development continuously strives to address the complex requirements of an ideal wound dressing. In this study, the same principles were followed, aligning the material design with the specific challenges posed by chronic wounds. In this context, the polymeric matrix represents the bulk of the dressing, while the therapeutic agents are incorporated in smaller quantities relative to the total material. The selection of alginate as the structural matrix offers several advantages, including excellent biocompatibility, intrinsic porosity, and the ability to incorporate and release therapeutic agents directly at the wound site. SEM micrographs of the five developed formulations confirm the porous nature of the hydrogels, as well as the successful incorporation of the therapeutic agents, which influences the overall morphology of the wound dressings.

Ag nanoparticles likely act as rigid inorganic fillers that hinder the formation of ice crystals and allow the pores to form into a continuous structure. In contrast, the incorporation of BC@PVP contributes to increased viscosity and softening of the alginate network, which results in a weaker structural framework around the pores and a more fragile morphology. The dual incorporation of both therapeutic agents appears to balance these opposing effects, leading to a more compact porous matrix. Notably, the addition of *Centella asiatica* extract does not alter the overall morphology of the hydrogel, which is advantageous in preserving the structural integrity while enhancing the therapeutic profile through botanical components.

The antimicrobial performance of the five alginate-based hydrogel formulations was assessed by measuring the diameter of the inhibition zone and quantifying the residual biofilm formation after 24 h of incubation with *S. aureus* and *E. coli*.

The antimicrobial performance observed in the developed dressings is consistent with the theoretical contributions of the incorporated therapeutic agents. The sample containing only Ag nanoparticles (ALG_Ag) exhibited the strongest inhibitory effect, as expected, given that Ag nanoparticles together with cinnamon oil are well known for their potent and broad-spectrum antimicrobial activity [90,91,92]. Their small size (17.21 ± 0.42 nm), confirmed by SEM, along with their high colloidal stability (zeta potential of –38.49 mV), ensures an effective interaction with bacterial membranes, leading to cell damage and inhibition of growth. In the case of the dual-loaded formulation ALG_Ag_BC_C, the highest antimicrobial efficiency was observed, suggesting a synergistic contribution from both Ag nanoparticles and *Centella asiatica* extract. *Centella asiatica* is recognized for its phytochemical content, particularly asiaticoside and madecassoside, which exhibit intrinsic antibacterial properties, especially against Gram-positive strains [93,94,95]. On the other hand, samples containing BC@PVP polymeric nanoparticles (ALG_BC and ALG_Ag_BC) also showed notable antimicrobial activity.

The biological assessment of novel wound dressings is critical to determine their cytocompatibility with skin-relevant cells, such as keratinocytes, prior to advanced studies and in vivo testing. The biological evaluation of the alginate-based wound dressing demonstrated that the presence of silver ions, while beneficial for antimicrobial purposes, exhibited a mild cytotoxic effect and induced oxidative stress in human keratinocytes. However, the incorporation of antioxidant and bioactive agents significantly ameliorated these adverse effects. The ALG_Ag_BC_C formulation, in particular, preserved cell viability, minimized LDH leakage, and maintained ROS levels comparable to or below the pristine alginate control. These findings emphasize the importance of a carefully balanced formulation when designing wound dressings, a formulation that leverages the antimicrobial efficacy of silver while incorporating protective agents to mitigate its cytotoxic effects. Several studies have similarly reported that silver nanoparticles or silver ions can be cytotoxic to human skin cells, highlighting the need for dose control and synergistic design strategies in the development process. In general, because Ag nanoparticles are often coated with organic or inorganic moieties to optimize their interactions with living cells while preserving antimicrobial activity [96], the same design principle underlies the dressings developed in this study. For example, in the ALG_Ag_BC_C formulation, the addition of *Centella asiatica* extract, which is rich in phenolic constituents capable of adsorbing onto Ag surfaces, can be regarded as a natural capping approach. Moreover, given the antioxidant capacity of *Centella asiatica* and its pro-angiogenic/pro-collagen effects mediated by madecassoside, co-loading with this extract is expected to mitigate silver-associated cytotoxicity; accordingly, LDH release and intracellular ROS levels were lower than in the Ag-only dressing [97,98]. Likewise, β-carotene nanoparticles provide complementary anti-inflammatory and antioxidant effects; their co-presence with Ag nanoparticles further reduced LDH and ROS, indicating a less stressed cellular response [99,100]. Therefore, potential negative effects triggered by silver can be counterbalanced through the strategic inclusion of bioactive compounds with antioxidant properties, potentially expanding the safety of silver-based dressings for wound healing applications [101,102,103,104].

## 3. Conclusions

This study reports the development of multifunctional alginate-based wound dressings incorporating silver nanoparticles, beta-carotene nanocarriers, and *Centella asiatica* extract. The hydrogels displayed porous, stable structures with high swelling capacity and effective antimicrobial activity against *Staphylococcus aureus* and *Escherichia coli*. Among all formulations, ALG_Ag_BC_C showed the best balance between antimicrobial performance and cytocompatibility, as beta-carotene and *Centella asiatica* mitigated silver-induced cytotoxicity and oxidative stress. These results highlight the potential of ALG_Ag_BC_C as a candidate for advanced wound care and warrant further preclinical validation. As a proof-of-concept study, this work opens several directions for further investigation. Future efforts will include detailed evaluation of β-carotene stability after processing, together with encapsulation efficiency, drug loading, and multi-batch reproducibility to confirm process robustness. In addition, in vivo validation will be pursued to substantiate the biological performance of the developed dressings in complex wound environments.

## 4. Materials and Methods

The materials used for the development of antimicrobial and regenerative wound dressings in this study include silver nitrate (AgNO_3_), sodium hydroxide (NaOH), D-glucose, cinnamon essential oil, polyvinylpyrrolidone (PVP), Tween 80, beta-carotene, ethanol, sodium alginate, *Centella asiatica* extract, and ultrapure water. The cinnamon essential oil was purchased from SC Solaris Plant SRL (Bucharest, Romania), the *Centella asiatica* extract was obtained from SC Dibra Connections SRL (Bucharest, Romania), and all other reagents were acquired from Sigma-Aldrich (St. Louis, MO, USA).

### 4.1. Synthesis of Silver Nanoparticles (Ag)

The chemical reduction of metallic salt, specifically silver nitrate, was utilized to synthesize Ag nanoparticles. Aqueous AgNO_3_ solution (1 mg/mL) served as the silver precursor in the synthesis process, and a 0.25 M sodium hydroxide solution was prepared using ultrapure water. Subsequently, 1 g of D-glucose was added to the alkaline solution, which was then heated to 80 °C under continuous stirring until a light-yellow coloration appeared. Finally, 500 μL of cinnamon essential oil was added, serving as a natural capping and stabilizing agent due to its high content of bioactive compounds such as cinnamaldehyde and eugenol. The first solution was slowly added dropwise to the organic phase under continuous magnetic stirring. Following synthesis, the resulting precipitate was collected by centrifugation, washed three times with ultrapure water to remove unreacted residues, and subsequently dried at room temperature.

### 4.2. Synthesis of Beta-Carotene-Loaded Polymeric Nanoparticles (BC@PVP)

Beta-carotene-loaded polymeric nanoparticles were prepared using PVP as the encapsulating polymer and Tween 80 as a surfactant. Initially, 100 μL of Tween 80 was dissolved in 50 mL of ethanol under continuous magnetic stirring to ensure uniform dispersion. To this solution, 1000 mg of PVP and 100 mg of beta-carotene were added, and the mixture was stirred at 60 °C for 30 min to facilitate solubilization. The nanoparticle formation was induced by probe sonication, applied in pulsed mode (30 s on, 10 s off) for a total duration of 3 min. This process enabled the encapsulation of beta-carotene within the polymeric matrix and promoted the formation of nanosized, stable particles dispersed in the ethanolic medium.

### 4.3. Formulation of Alginate-Based Hydrogels (ALG, ALG_Ag, ALG_BC, ALG_Ag_BC, ALG_Ag_BC_C)

For the preparation of the dressings, a 5% (*w*/*v*) sodium alginate solution was prepared by dissolving the polymer in ultrapure water under continuous stirring until a homogeneous gel was obtained. The solution was then evenly divided into five containers, each further formulated by incorporating silver nanoparticles (AgNPs) and/or beta-carotene-loaded polymeric nanoparticles (BC@PVP), depending on the desired composition.

The first sample served as the control formulation, containing only the alginate matrix without any added therapeutic agents, and is referred to hereafter as ALG. The second formulation involved the addition of silver nanoparticles to the ALG matrix at a final concentration of 1 mg/mL, and was designated as ALG_Ag.

For the third formulation, BC@PVP nanoparticles were added to the alginate matrix to achieve a final concentration of 2 mg/mL, and this sample was labeled ALG_BC. The fourth sample, ALG_Ag_BC, was obtained by incorporating both therapeutic agents (Ag and BC@PVP) into the alginate matrix at the same concentrations used in the individual formulations.

To further enhance the bioactivity of the dressings, particularly in line with the green synthesis principles applied throughout this study, a botanical extract of *Centella asiatica* was added as a regenerative booster. The final formulation, named ALG_Ag_BC_C, was prepared identically to ALG_Ag_BC but included approximately 1% (*v*/*v*) *Centella asiatica* extract relative to the alginate matrix.

**Figure 18:**
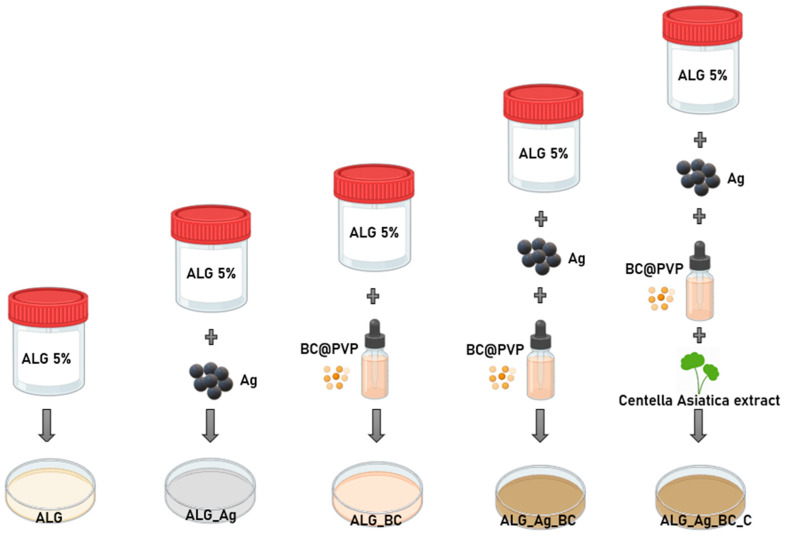
Schematic representation of each formulation composition.

All samples were frozen for 24 h, followed by lyophilization process for 72 h to obtain the final porous hydrogel dressings. Figure 18 provides a schematic representation of wound dressings’ composition.

### 4.4. Investigation Methods

The X-ray Diffraction (XRD) technique was used to investigate the phase and crystalline parameters of silver nanoparticles. A PANalytical Empyrean model was operated to perform the analysis purchased from PANalytical (Almelo, The Netherlands) featuring a hybrid monochromator (2xGe 220) on the incident side and a parallel plate collimator found on the PIXcel 3D detector on the diffracted side. Grazing Incidence XRD (GIXRD) mode was applied at ambient temperature, with a fixed incidence angle (ω) of 0.5°, and the 2θ scan range set between 10° and 80°. The system operated using Cu Kα radiation (λ = 1.5406 Å), at 45 kV and 40 mA.

Fourier-transform infrared (FTIR) spectroscopy was employed to identify the functional groups present in the BC@PVP polymeric nanoparticles as well as in the developed wound dressing formulations. The analysis was carried out using a Thermo iN10-MX spectrometer (Thermo Fisher Scientific, Waltham, MA, USA) equipped with a ZnSe crystal. Spectra were recorded in absorbance mode, with a background spectrum collected prior to each sample measurement. The instrument was set to a resolution of 8 cm^−1^, with 64 scans per spectrum, over a spectral range of 4000–400 cm^−1^.

Morphological analysis, as well as particle and pore size evaluation, were carried out using scanning electron microscopy (SEM). The investigations were performed with an Inspect F50 SEM system (FEI, Hillsboro, OR, USA), equipped with an energy-dispersive X-ray spectroscopy (EDS) module for elemental characterization. Micrographs were acquired using both secondary electron and backscattered electron detectors, at an accelerating voltage of 30 keV. For Ag nanoparticles, a small amount of dry powder was evenly dispersed on a carbon adhesive tape mounted on an SEM stub. In the case of BC@PVP nanoparticles, 10 µL of the suspension was deposited onto the carbon tape and allowed to gradually air-dry at room temperature. The lyophilized wound dressings were cut into 5 mm discs and mounted directly onto the carbon tape before being introduced into the SEM analysis chamber.

The physicochemical behavior of the nanoparticles in liquid suspension was assessed using dynamic light scattering (DLS). Measurements were performed with a DelsaMax Pro instrument (Beckman Coulter, Brea, CA, USA), equipped with a 532 nm laser. Samples were dispersed in ultrapure water and subjected to ultrasonic treatment for 10 min in a water bath prior to analysis, to ensure uniform dispersion and minimize aggregation.

To assess the swelling capacity, lyophilized dressings were cut into cylindrical shapes with a diameter of 5 mm. Each sample was immersed in 5 mL of ultrapure water at room temperature. The time-dependent swelling ratio was calculated using the following formula:

$$Swelling\;ratio=\frac{W_{t}-W_{i}}{W_{i}}\times 100\%$$

where *W_i_* is the initial dry weight of the hydrogel, and *W_t_* is the weight after immersion at specific time intervals.

The initial stage of dressings degradation was also investigated, as it represents a critical parameter for wound dressing performance. The degradation rate was determined based on the following equation:

$$Degradation\;=\;(1\;-\;\frac{W_{0}-W_{t}}{W_{0}})\;\times \;100\%$$

where *W*_0_ is the initial dry weight of the hydrogel before immersion, and *W_t_* is the dry weight of the sample recovered at defined time points after liquid incubation.

### 4.5. Antimicrobial Evaluation

#### 4.5.1. Bacterial Strains

To evaluate the antibacterial performance of the developed wound dressings, two clinically relevant bacterial strains commonly associated with chronic wound infections were selected. The assay included one Gram-positive strain, *Staphylococcus aureus* ATCC 25,923, and one Gram-negative strain, *Escherichia coli* ATCC 25,922. Both strains are widely recognized as opportunistic pathogens frequently isolated from infected wounds and are standard reference strains for antimicrobial susceptibility testing.

#### 4.5.2. Adapted Disc Diffusion Protocol 

The evaluation of antibacterial activity for alginate-based dressings was performed using a modified disc diffusion method, adapted to the physical and chemical nature of hydrogels, in accordance with CLSI recommendations (2021). The procedure began with the preparation of a standardized microbial inoculum, consisting of a suspension of bacterial cells in sterile physiological saline, derived from 18–24-h cultures. The cell density was adjusted to 0.5 McFarland standard (approximately 1.5 × 10^8^ CFU/mL). These cultures were obtained by inoculating the tested bacterial strains onto solid nutrient agar. Nutrient agar (pH 7.2–7.4) was poured to a uniform thickness of 4 mm into 9 cm Petri dishes and allowed to solidify at room temperature. The surface of each agar plate was then uniformly inoculated by swabbing with the prepared bacterial suspension using a sterile cotton swab. Hydrogel discs were aseptically placed directly onto the inoculated agar surfaces. The plates were incubated at 37 °C for 24 h. After incubation, the diameter of the inhibition zones was measured. The size of the inhibition zone was used as an indicator of antibacterial activity, with larger diameters reflecting greater antimicrobial effectiveness of the hydrogel formulation.

#### 4.5.3. Monospecific Biofilm Development

The antibiofilm activity of the wound dressings was assessed using an in vitro monospecific biofilm model. This assay was designed to evaluate the ability of each formulation to inhibit or reduce bacterial biofilm formation. Circular samples (5 mm diameter) were sterilized by UV irradiation (20 min per side) and placed into sterile 24-well plates, each containing 1 mL of nutrient broth. Subsequently, 10 μL of bacterial suspension (adjusted to 0.5 McFarland standard) from *S. aureus* or *E. coli* was added to each well. The samples were incubated at 37 °C for 24 h to allow initial bacterial adhesion. After incubation, the culture medium was removed, and each sample was gently washed with sterile phosphate-buffered saline (PBS) to eliminate planktonic cells. The dressings were transferred to new wells containing fresh sterile broth and incubated for another 24 h to promote mature biofilm development. After incubation, the hydrogel discs were then transferred into 1.5 mL microcentrifuge tubes containing 1 mL of fresh sterile PBS. The tubes were vortexed for 30 s and subjected to 10 s of ultrasonication to detach biofilm-associated bacteria from the hydrogel surface. The resulting suspensions were serially diluted (10-fold) and plated on nutrient agar to determine the number of viable bacteria within the biofilm, expressed as colony-forming units per milliliter (CFU/mL).

### 4.6. In Vitro Biological Evaluation

#### 4.6.1. Cell Culture and Experimental Design

To evaluate the biocompatibility of the developed wound dressings, the human keratinocyte cell line HaCaT was employed as in vitro model. Cells were cultured in Dulbecco’s Modified Eagle Medium (DMEM, Sigma Aldrich, St. Louis, MO, USA), supplemented with 10% fetal bovine serum (FBS, Gibco, Thermo Fisher Scientific, Waltham, MA, USA) and 1% Penicillin/Streptomycin (P/S, Sigma Aldrich), under standard culture conditions (37 °C, 5% CO_2_, 95% humidity).

Prior to cell seeding, the wound dressing samples were sterilized by exposure to ultraviolet (UV) light for 20 min. HaCaT cells were seeded directly onto the sterilized dressings at a density of 1.5 × 10^4^ cells/cm^2^ to generate material-cells bioconstructs. These bioconstructs were cultured for 72 h under standard incubation conditions, with analyses performed at 24 and 72 h post-seeding.

#### 4.6.2. Biocompatibility Assessment

Cell metabolic activity was evaluated using the MTT assay as an indicator of cell viability post-contact with the novel wound dressings. After removing the culture medium, cells were incubated with 1 mg/mL MTT solution (3-(4,5-dimethilthiazol-2-il)-2,5-dipheniltetrazolium, Sigma Aldrich) freshly prepared in serum-free DMEM, for 4 h at 37 °C. Following incubation, the resulting formazan crystals were solubilized with 2-propanol, and the absorbance was measured at 550 nm using a FlexStation 3 multimodal reader (Molecular Devices, San Jose, CA, USA). Cell viability was expressed as a percentage relative to the untreated control group at 24 h, which was set at 100%.

To assess membrane integrity and cytotoxic potential, lactate dehydrogenase (LDH) release into the culture medium was measured. Cell culture medium samples were collected and analyzed using In vitro Toxicology TOX-7 assay kit (Sigma Aldrich), according to the manufacturer’s instructions. After a 30 min incubation with the kit components, the reaction was stopped by the addition of HCl, and absorbance was measured at 490 nm using the Flex Station 3.

Intracellular reactive oxygen species (ROS) levels were quantified using the fluorescent probe DCFH-DA (2′, 7′-dichlorofluorescein diacetate, Sigma-Aldrich). For this purpose, bioconstructs were washed with phosphate-buffered saline (PBS) and incubated with 10 μM DCFH-DA diluted in serum-free DMEM for 30 min at 37 °C in the dark. Excess dye was removed by PBS washing, and fluorescence was measured immediately at excitation/emission wavelengths of 485/530 nm using a FlexStation 3 microplate reader. ROS levels were expressed relative to the untreated control according to the following formula: ROS Production (%) = (Fluorescence of treated cells/Fluorescence of control cells) × 100.

All experiments were performed in triplicate and data are presented as mean ± standard deviation (SD) of three independent experiments. Statistical analysis was conducted using GraphPad Prism Version 9.3.0, with significance set at *p* ≤ 0.05.

## Figures and Tables

**Table 1:** FTIR data compiled in tabular form.

Functional Group	Wavenumber (cm^−1^)	References
(PVP) O–H stretching vibration	3420 cm^−1^	[65,66,67]
(β-Carotene) C–H stretch (aliphatic)	2950 cm^−1^	[68,69,70]
(Tween 80) C–H stretch	2923 cm^−1^	[71]
(PVP) C=O stretching vibration	1647 cm^−1^	[65,66,67]
(β-Carotene) C=C stretch conjugated	1558 cm^−1^	[68,69,70]
(β-Carotene) CH_2_ scissor band/CH_3_ bend	1436 cm^−1^	[68,69,70]
(PVP) C–N vibration	1287 cm^−1^	[65,66,67]
(Tween 80) C–O–C ether stretching	1096 cm^−1^	[71]
(PVP) C–H wagging/ring deformation (pyrrolidone)	844 cm^−1^	[65,66,67]
(Tween 80) C–H wagging (aliphatic CH_2_ in ethoxylated segments)	844 cm^−1^	[71]
(β-Carotene) Skeletal deformation of polyene chain	571 cm^−1^	[68,69,70]

## Data Availability

The raw data supporting the conclusions of this article will be made available by the authors on request.
